# SpaICL: image-guided curriculum strategy-based graph contrastive learning for spatial transcriptomics clustering

**DOI:** 10.1093/bib/bbaf433

**Published:** 2025-08-21

**Authors:** Jingcheng Zhao, Wenwen Min

**Affiliations:** School of Information Science and Engineering, Yunnan University, 650500, Yunnan, China; School of Information Science and Engineering, Yunnan University, 650500, Yunnan, China

**Keywords:** spatial transcriptomics, histological image, domain identification, multimodal integration

## Abstract

Spatial transcriptomics, by capturing both gene expression and spatial information, holds great promise for unraveling the complex organization of tissues. In this study, we introduce SpaICL, an image-guided curriculum strategy-based graph contrastive learning framework for spatial transcriptomics clustering. SpaICL integrates gene expression, spatial coordinates, and histological image features to construct a low-dimensional latent representation that enhances the de-lineation of spatial functional domains. The model employs a complementary masking strategy and a shared graph neural network encoder to generate dual embeddings, while a dual cross-attention mechanism aligns local and global features across multiple modalities. Additionally, the curriculum learning module further facilitates the gradual integration of neighborhood information, effectively mitigating the over-smoothing issues associated with fixed adjacency matrices. We evaluated the performance of SpaICL on five benchmark spatial transcriptomics datasets, achieving superior results compared to existing baseline methods. Moreover, SpaICL demonstrates significant potential in downstream analytical applications. The code of SpaICL is available at https://github.com/wenwenmin/SpaICL.

## Introduction

The spatial context of gene expression plays a pivotal role in regulating cellular identity, behavior, and interactions within tissues. Spatial transcriptomics (ST) technologies, such as 10$\times $ Genomics Visium (https://www.10xgenomics.com/), empower researchers to analyze gene expression with spatial resolution, while simultaneously capturing high-resolution histological images. This integration offers unprecedented insights into tissue architecture, developmental dynamics, and disease microenvironments. A central computational task in ST data analysis is clustering, which aims to delineate spatial domains characterized by coherent molecular features. However, effectively integrating spatial information with high-dimensional gene expression data remains a major computational challenge [1–3].

Early clustering methods for ST data often relied on conventional non-spatial algorithms, such as K-means and Louvain [4]. While computationally efficient, these approaches treat each spot independently and ignore spatial continuity, often failing to capture biologically meaningful tissue structures.

To address these limitations, spatially aware clustering algorithms have been proposed [5]. Notable methods like DiffusionST [6], STAGATE [7], and SEDR [8] incorporate spatial information to guide representation learning. For instance, STAGATE leverages graph attention mechanisms to model neighborhood dependencies; DiffusionST enhances spatial signal propagation using a diffusion-based approach with zero-inflated negative binomial (ZINB) modeling; and SEDR employs variational graph autoencoders (VGAE) to combine spatial embeddings. However, these methods primarily focus on transcriptomic modeling and often struggle to resolve complex spatial patterns, especially in heterogeneous or morphologically diverse regions. Moreover, they tend to under explore the potential of contrastive learning for capturing discriminative spatial representations.

Recent advances such as GraphST [9] and CCST [10] incorporate contrastive learning to improve representation learning [11]. GraphST generates multiple data views through random masking and projection, while CCST employs Deep Graph Infomax (DGI) to maximize mutual information between global and local representations. Despite their improvements in spatial representation, these methods mainly focus on gene expression features and overlook complementary modalities such as tissue morphology. Additionally, their view construction strategies often lack semantic diversity, which can limit the strength and generalizability of the contrastive signal.

Some approaches have attempted to incorporate histological image features [12, 13] into spatial clustering. For example, DeepST [14] and SpaGCN [15] utilize tissue images to refine spatial graphs or guide representation learning. However, their fusion strategies are often shallow or indirect. SpaGCN uses a single weighted RGB combination to represent histological information, a linear dimensionality reduction that fails to capture complex texture, morphology, and cellular structures—potentially discarding critical microenvironmental cues necessary for fine-grained spatial delineation. DeepST relies on ResNet to extract morphological features but only uses them to modify the adjacency matrix, missing opportunities for deep integration with gene expression embeddings. Furthermore, these models lack dynamic cross-modal alignment and often overlook the differing complexities in learning local versus global structures during training.

**Figure 1:**
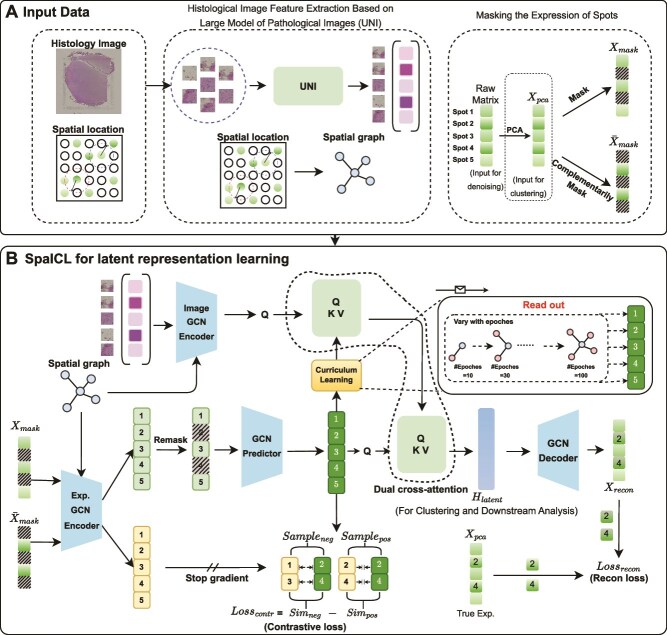
Overview of SpaICL. (A) Histology images are partitioned into patches based on spot coordinates and then the features of each spot image are subsequently extracted using the UNI model. Meanwhile, Euclidean distances between spots are computed to construct the adjacency matrix. (left) The raw matrix is reduced via PCA and then masked to obtain a masked matrix and a complementary masked matrix. (right) (B) The image features and complementary masked matrices are encoded via a GCN, then fused using the Curriculum Learning Module (CLM) and Dual Cross-Attention to extract the final latent embedding. CLM progressively reads out information from more neighboring nodes as training iterations proceed, leading to a more robust learning state.

To overcome these limitations, we propose SpaICL, a novel image-guided curriculum strategy-based graph contrastive learning framework for spatial transcriptomics clustering (Fig. 1). SpaICL introduces several key innovations: (1) a complementary masking strategy to generate informative gene expression views, enriching contrastive representation learning; (2) the use of the pathology foundation model UNI [16] to extract domain-aware, high-level features from histological images, surpassing traditional architectures in morphological understanding; (3) a dual cross-attention fusion module to deeply integrate morphological features from histological images and gene embeddings, enabling more precise multimodal fusion; and (4) a curriculum learning paradigm that progressively guides the model from local feature alignment to global structure learning, enhancing training stability and multiscale sensitivity. Together, these innovations enable SpaICL to produce robust, biologically meaningful low-dimensional embeddings, facilitating accurate spatial domain identification across diverse datasets.

We evaluated SpaICL on four 10x Genomics Visium ST datasets and one Visium-like ST dataset, conducting comprehensive benchmarking against state-of-the-art methods. The results demonstrate that SpaICL consistently achieves superior performance in spatial transcriptomics clustering, underscoring its effectiveness for downstream analytical tasks.

## Materials and methods

### Overview of the SpaICL workflow

The proposed SpaICL framework seamlessly integrates gene expression profiles, spatial coordinates, and tissue morphology to construct a unified low-dimensional latent representation, enabling accurate identification of spatial domains (see Fig. 1). It first applies a complementary masking strategy to gene expression data, producing two embeddings via a shared graph neural network. Histological images are processed by a pretrained large model of pathological images (UNI) [16] to extract morphological features, projected into the same space. A dual-layer cross-attention module aligns and fuses modalities, aided by a curriculum learning strategy for stable training. The final representation supports downstream tasks such as domain identification and visualization. The detailed algorithmic process is shown in Algorithm 1.


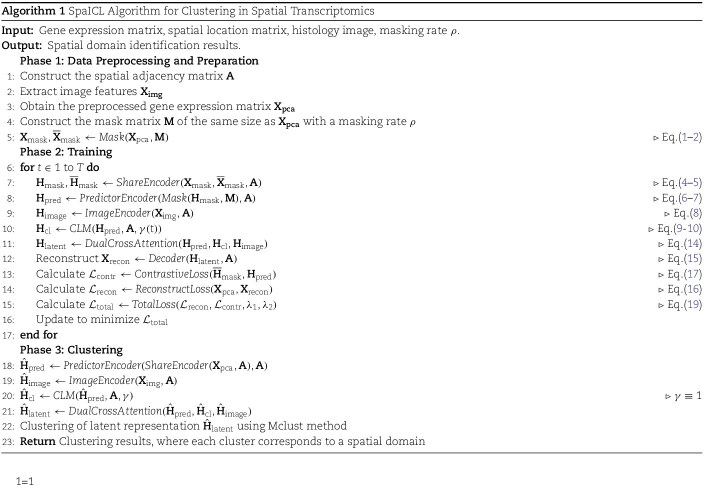


### Datasets

This study utilizes five spatial transcriptomics (ST) datasets (Table 1). The DLPFC [17] (human dorsolateral prefrontal cortex), BRCA [8] (human breast cancer), MBA [18] (anterior mouse brain tissue), and BCDC [19] (human breast cancer: ductal carcinoma in situ and invasive carcinoma) datasets were obtained from the 10x Visium platform, whereas the HER2+ [20] (HER2-positive breast tumors) dataset originates from a Visium-like plat-form.

**Table 1:** Summary of the ST datasets used in this study

**Datasets**	**Slices**	**Spots**	**Genes**	**Domains**	**Ref.**
DLPFC	12	3460–4789	33538	5–7	[17]
BRCA	1	3798	36601	20	[8]
MBA	1	2695	32285	52	[18]
HER2+	8	176–613	14532–15301	4–7	[20]
BCDC	1	2518	17651	2	[19]

The DLPFC dataset consists of 12 slices collected from three individuals, with each individual contributing four slices. The number of spots per slice ranges from 3460 to 4789, and spatial domains were manually annotated by the original authors, covering 5–7 regions. The HER2+ dataset contains eight slices, each derived from a different individual’s HER2+ tumor, with spot counts ranging from 176 to 613 and spatial domains annotated into 4–7 categories. The BRCA, MBA, and BCDC datasets are single-slice datasets, BRCA was annotated into 20 spatial domains by Xu *et al.* [8], with 3798 spots. MBA was manually labeled into 51 known clusters and one unmarked region, comprising 2695 spots. BCDC was classified into tumor and non-tumor domains, containing 2518 spots.

### Data preprocessing and preparation

#### Spatial graph construction

To construct the spatial graph, we compute the Euclidean distance between each pair of spots based on their spatial coordinates. Using the k-nearest neighbors (KNN) algorithm, we assign each spot to its $K$ closest neighbors, forming an adjacency matrix $\mathbf{A} \in \mathbb{R}^{N \times N}$. If spot $j$ is a neighbor of spot $i$, then $A_{ij} = A_{ji} = 1$; otherwise, $A_{ij} = 0$ (Fig. 1A(left)).

#### Image feature extraction

To incorporate histological image features, we extract image patches centered around each spot using its spatial coordinates $(x_{i}, y_{i})$. Each patch is cropped with a size matching the spot diameter (e.g. 177.48 $\times $ 177.48 pixels for BRCA) as provided by the dataset specifications. These patches, obtained from H&E-stained histology images, are resized to 224 $\times $ 224 pixels using torchvision.transforms.Resize and normalized by mean [0.485, 0.456, 0.406] and standard deviation [0.229, 0.224, 0.225] via transforms.Normalize. Subsequently, we employ the UNI [16], a general-purpose self-supervised model for pathology, to extract deep image features, resulting in an image feature matrix $\mathbf{X}_{\text{img}} \in \mathbb{R}^{N \times 1024}$, where $N$ is the total number of spots (Fig. 1A(left)).

#### Gene expression data processing

We first filter out genes and spots with no detectable expression in the dataset. Next, we apply log normalization to the entire expression matrix using the SCANPY Python library. To select the most informative genes, we retain only the top $N_{h}$ highly variable genes, forming the gene expression matrix $\mathbf{X}_{\text{ori}} \in \mathbb{R}^{N \times N_{h}}$, where $N$ represents the total number of spots. Finally, we apply Principal Component Analysis (PCA) to reduce the dimensionality of the gene expression data, selecting the top $N_{\text{pca}}$ principal components (PCs) as the primary input for our model, resulting in $\mathbf{X}_{\text{pca}} \in \mathbb{R}^{N \times N_{\text{pca}}}$ (Fig. 1A(right)).

#### Complementary masking strategy

In spatial transcriptomics analysis, gene expression matrices are often high-dimensional, sparse, and noisy, which can lead to overfitting or sensitivity to outliers. To enhance model robustness and generalization, we introduce a *Complementary Masking* strategy as a data augmentation method.

We randomly mask a subset of spots in $\mathbf{X}_{\text{pca}}$ with a predefined masking ratio $\rho $, setting their expression values to zero. This results in the masked dataset $\mathbf{X}_{\text{mask}}$, defined as 


(1) \begin{align*}& \mathbf{X}_{\text{mask}} = \mathbf{X}_{\text{pca}} \odot \mathbf{M},\end{align*}

where $\odot $ represents the Hadamard product, and $\mathbf{M}$ is a binary mask matrix containing values of 0s and 1s. Simultaneously, we generate a complementary masked dataset $\overline{\mathbf{X}}_{\text{mask}}$, defined as 


(2) \begin{align*}& \overline{\mathbf{X}}_{\text{mask}} = \mathbf{X}_{\text{pca}} \odot (1 - \mathbf{M}).\end{align*}

This ensures that the non-masked regions in $\mathbf{X}_{\text{mask}}$ correspond to the masked regions in $\overline{\mathbf{X}}_{\text{mask}}$, satisfying the condition (Fig. 1A(right)): $\mathbf{X}_{\text{mask}} + \overline{\mathbf{X}}_{\text{mask}} = \mathbf{X}_{\text{pca}}$ and $\mathbf{X}_{\text{mask}} \odot \overline{\mathbf{X}}_{\text{mask}} = \mathbf{0}$.

### Feature encoding and latent representation learning

The proposed model incorporates multiple Graph Convolutional Network (GCN) encoding modules to comprehensively capture both global and local characteristics from spatial transcriptomics (ST) data (gene expression matrices) and histopathological image features. These modules facilitate the learning of low-dimensional latent representations. Specifically, we design three key components: Masked Data Encoder, Image Feature Encoder, and Predictor (Fig. 1B).

GCN operates by aggregating information from neighboring nodes in a graph, updating each node’s representation based on its local topology. The feature update process in each GCN layer is expressed as: 


(3) \begin{align*}& \mathbf{H}^{(l+1)} = \mathcal{F}_{GCN}(\mathbf{H}^{(l)}, \mathbf{A}) = \sigma\left( \tilde{\mathbf{D}}^{-\frac{1}{2}} \tilde{\mathbf{A}} \tilde{\mathbf{D}}^{-\frac{1}{2}} \mathbf{H}^{(l)} \mathbf{W}^{(l)} \right)\end{align*}

where $\tilde{\mathbf{A}} = \mathbf{A} + \mathbf{I}$ denotes the adjacency matrix with self-loops added, $\tilde{\mathbf{D}}$ is the degree matrix of $\tilde{\mathbf{A}}$, $\mathbf{H}^{(l)}$ represents the feature matrix at layer $l$, $\mathbf{W}^{(l)}$ is a learnable weight matrix, and $\sigma $ is a non-linear activation function. The normalization step prevents imbalances due to differences in node degrees, stabilizing model training.

#### Masked data encoding

For both primary masked data and complementary masked data, we employ a shared GCN encoder (denoted as $\text{GCN}_{\text{share}}$) to extract feature representations: 


(4) \begin{align*} & \mathbf{H}_{\text{mask}} = \text{GCN}_{\text{share}}(\mathbf{X}_{\text{mask}}, \mathbf{A}), \end{align*}

(5) \begin{align*} & \overline{\mathbf{H}}_{\text{mask}} = \text{GCN}_{\text{share}}(\overline{\mathbf{X}}_{\text{mask}}, \mathbf{A}) \end{align*}

where $\mathbf{H}_{\text{mask}}$ and $\overline{\mathbf{H}}_{\text{mask}}$ are the latent representations of the primary masked data and complementary masked data, respectively. The complementary masked representation $\overline{\mathbf{H}}_{\text{mask}}$ is further used to construct positive and negative samples for contrastive learning.

#### Latent representation

To reduce redundancy and ensure accurate representation of spatial features, we apply a remasking operation on $\mathbf{H}_{\text{mask}}$, producing $\mathbf{H}_{\text{remask}}$: 


(6) \begin{align*}& \mathbf{H}_{\text{remask}} = \mathbf{H}_{\text{mask}} \odot \mathbf{M}\end{align*}

where $\mathbf{M}$ is the same binary mask matrix used to generate the primary masked data. Then, a GCN-based predictor $\text{GCN}_{\text{predictor}}$ encodes $\mathbf{H}_{\text{remask}}$ to obtain $\mathbf{H}_{\text{pred}}$, enhancing spatial information modeling: 


(7) \begin{align*}& \mathbf{H}_{\text{pred}} = \text{GCN}_{\text{predictor}}(\mathbf{H}_{\text{remask}}, \mathbf{A})\end{align*}

The objective of this step is to make $\mathbf{H}_{\text{pred}}$ approximate the low-dimensional representation of the complete data while leveraging spatial structures to fill in missing information. $\mathbf{H}_{\text{pred}}$ is also used in contrastive loss computation.

#### Histology feature encoding

Since we have incorporated spatial structure in gene expression modeling, we aim to jointly model histopathological image features instead of treating them independently. To achieve this, we use a GCN-based image feature encoder $\text{GCN}_{\text{image}}$ to encode the extracted image features $\mathbf{X}_{\text{img}}$, yielding the latent image representation $\mathbf{H}_{\text{image}}$: 


(8) \begin{align*}& \mathbf{H}_{\text{image}} = \text{GCN}_{\text{image}}(\mathbf{X}_{\text{img}}, \mathbf{A})\end{align*}

### Modality fusion

#### Curriculum learning module

The curriculum learning [21] module (CLM) dynamically extracts information during training. As training epochs increase, this module progressively selects a certain percentage ($\gamma $) of a spot’s spatial neighbors for fusion with image features. The percentage of selected neighbors increases as training progresses, ensuring a gradual transition from local to global feature integration. The function governing $\gamma $ is defined as: 


(9) \begin{align*}& \gamma = \begin{cases} \frac{\epsilon_{\text{cur}}}{\epsilon_{\text{total}}}, & \text{if}\ \frac{\epsilon_{\text{cur}}}{\epsilon_{\text{total}}} < 0.9 \\ 1, & \text{if}\ \frac{\epsilon_{\text{cur}}}{\epsilon_{\text{total}}} \geq 0.9 \end{cases}\end{align*}

where $\epsilon _{\text{total}}$ is the total number of training epochs, and $\epsilon _{\text{cur}}$ is the current epoch, the detailed procedure is illustrated in (Supplementary Figure S1). For each spot $i$, a subset of neighbors ($\gamma $ proportion) is randomly selected from $\mathbf{H}_{\text{pred}}$, forming: 


(10) \begin{align*}& \mathbf{H}^{i}_{\text{cl}} = \mathcal{R}(\text{rand}(\mathbf{H}_{\text{neighbor}}^{i}, \gamma))\end{align*}

where $\mathbf{H}_{\text{neighbor}}^{i}$ represents all neighboring spots of spot $i$ in $\mathbf{H}_{\text{pred}}$, the operator $\text{rand}(\mathbf{H}_{\text{neighbor}}^{i}, \gamma )$ randomly samples a subset from $\mathbf{H}_{\text{neighbor}}^{i}$ with sampling ratio $\gamma $, and $\mathcal{R}(\cdot )$ denotes the readout [22] function. This randomized selection ensures that the model does not overly rely on specific neighbors, enhancing robustness. Additionally, the model reserves the final 10% of training epochs for complete global modality fusion. After training, $\gamma $ is fixed at 1 and no longer changes.

#### Dual cross-attention for multimodal fusion

Given the success of cross-attention [23] mechanisms in multimodal learning, we introduce a Dual Cross-Attention (DCA) module to integrate spatial transcriptomics and histology features. The cross-attention mechanism is defined as: 


(11) \begin{align*}& \text{CrossAttention}(\mathbf{Q}, \mathbf{K}, \mathbf{V}) = \text{softmax}\left( \frac{\mathbf{Q} \mathbf{K}^\top}{\sqrt{d_{k}}} \right) \mathbf{V}\end{align*}

where $\mathbf{Q}$ (query) comes from the target modality, $\mathbf{K}$ and $\mathbf{V}$ (key and value) come from the source modality, $d_{k}$ is the feature dimension of $\mathbf{K}$, and $\text{softmax}(\cdot )$ normalizes attention weights. This mechanism enables bidirectional information flow between different modalities.

#### Multi-head attention enhancement

To improve feature extraction, we employ multi-head attention, where multiple attention mechanisms operate in parallel: 


(12) \begin{align*}& \text{MultiHead}(\mathbf{Q}, \mathbf{K}, \mathbf{V}) = \text{Concat}(\text{head}_{1}, \dots, \text{head}_{h}) \mathbf{W}^{O}\end{align*}

where $\mathbf{W}^{O}$ is a learnable projection matrix, and each attention head is computed as: 


(13) \begin{align*}& \text{head}_{i} = \text{CrossAttention}(\mathbf{Q} \mathbf{W}_{i}^{Q}, \mathbf{K} \mathbf{W}_{i}^{K}, \mathbf{V} \mathbf{W}_{i}^{V})\end{align*}

where $\mathbf{W}_{i}^{Q}$, $\mathbf{W}_{i}^{K}$, and $\mathbf{W}_{i}^{V}$ are learnable transformation matrices for each attention head. Multi-head attention captures diverse feature subspaces, improving representation capability.

#### Final latent representation

Using DCA, we derive the latent representation $\mathbf{H}_{\text{latent}}$: 


(14) \begin{align*}& \mathbf{H}_{\text{latent}} = \text{MultiHead}(\mathbf{H}_{\text{pred}}, \tilde{\mathbf{H}}_{\text{image}}, \tilde{\mathbf{H}}_{\text{image}})\end{align*}

where $\tilde{\mathbf{H}}_{\text{image}} = \text{MultiHead}(\mathbf{H}_{\text{image}}, \mathbf{H}_{\text{cl}}, \mathbf{H}_{\text{cl}}).$ After training, $\mathbf{H}_{\text{latent}}$ is used for spatial domain identification and visualization in downstream analyses.

### Latent representation decoding

Instead of employing a symmetric autoencoder structure, we utilize a single-layer GCN decoder, denoted as $\text{GCN}_{\text{decoder}}$ (Fig. 1B), to map the latent representation $\mathbf{H}_{\text{latent}}$ back to the same representation space as $\mathbf{X}_{\text{pca}}$, thereby reconstructing the gene expression matrix $\mathbf{X}_{\text{recon}} \in \mathbb{R}^{N \times N_{\text{pca}}}$: 


(15) \begin{align*}& \mathbf{X}_{\text{recon}} = \text{GCN}_{\text{decoder}}(\mathbf{H}_{\text{latent}}, \mathbf{A})\end{align*}

### Loss function

#### Reconstruction loss

The reconstruction process aims to map the learned latent representations back to the original space while ensuring consistency with the original matrix. Given the high-dimensional and sparse nature of the original data, we adopt the Scaled Cosine Error (SCE) as the reconstruction objective function. Given a predefined scaling factor $\alpha $, the loss function is defined as: 


(16) \begin{align*}& \mathcal{L}_{\text{recon}} = \mathcal{L}_{\text{sce}} = \frac{1}{|V|} \sum_{i \in \mathcal{B}_{\text{mask}}} \left( 1 - \frac{\mathbf{x}_{i}^\top \mathbf{z}_{i}}{\|\mathbf{x}_{i}\| \cdot \|\mathbf{z}_{i}\|} \right)^\alpha, \quad \alpha \geq 1\end{align*}

where $\mathbf{x}_{i}$ and $\mathbf{z}_{i}$ correspond to the feature vectors in $\mathbf{X}_{\text{pca}}$ and $\mathbf{X}_{\text{recon}}$, respectively, and $|V|$ denotes the number of spots in $\mathcal{B}_{\text{mask}}$. In our model, $\alpha $ is fixed at 2 to reduce penalties on easily predictable samples and prevent overfitting on simple patterns.

#### Contrastive loss

Contrastive learning is a self-supervised learning paradigm that aims to learn effective feature representations by bringing similar sample pairs closer and pushing dissimilar pairs farther apart in the embedding space. Inspired by this principle, we design a contrastive loss to enhance the consistency between the masked features and their corresponding predictions.

We utilize $\mathbf{\overline{H}}_{\text{mask}}$ and $\mathbf{H}_{\text{pred}}$ from subsection 2.4 to construct positive and negative sample pairs. Given the complete set of spatial spots $\mathcal{B}$, we obtain the masked spot set $\mathcal{B}_{\text{mask}} \subset \mathcal{B}$ and the unmasked spot set $\mathcal{\overline{B}}_{\text{mask}} \subset \mathcal{B}$ according to the masking matrix $M$, such that $\mathcal{B}_{\text{mask}} \cup \overline{\mathcal{B}}_{\text{mask}} = \mathcal{B}$ and $\mathcal{B}_{\text{mask}} \cap \overline{\mathcal{B}}_{\text{mask}} = \varnothing $. From this, we define the positive samples as $S_{\text{pos}} = (A_{i}, B_{i})$ and the negative samples as $S_{\text{neg}} = (A_{j}, B_{k})$, where $A \in \overline{\mathbf{H}}_{\text{mask}}$, $B \in \mathbf{H}_{\text{pred}}$, $i \in \mathcal{B}_{\text{mask}}$, and $j, k \in \mathcal{B}$ with $j \neq k$. Since the number of negative samples greatly exceeds that of positive samples, we randomly select a subset of negative samples such that their number matches that of the positive pairs when constructing the positive and negative sample pairs $(S_{\text{pos}}, S_{\text{neg}})$. The contrastive loss $\mathcal{L}_{\text{contr}}$ is formulated by maximizing the similarity between positive pairs while minimizing the similarity of negative pairs: 


(17) \begin{align*}& \mathcal{L}_{\text{contr}} = \sum_{i=1}^{|N_{\mathcal{B}_{\text{m}}}|} \left( \text{Sim}_{\text{neg}}(S_{\text{neg}}^{i}) \right) - \sum_{j=1}^{|N_{\mathcal{B}_{\text{m}}}|} \left(\text{Sim}_{\text{pos}}(S_{\text{pos}}^{j}) \right)\end{align*}

where $|N_{\mathcal{B}_{\text{m}}}|$ denotes the number of spots in the masked set $\mathcal{B}_{\text{mask}}$. The cosine similarity is computed as follows: 


(18) \begin{align*}& \text{Sim}(\mathbf{A}, \mathbf{B}) = \frac{\mathbf{A} \cdot \mathbf{B}}{\|\mathbf{A}\| \times \|\mathbf{B}\|} = \frac{\sum_{i=1}^{n} A_{i} B_{i}}{\sqrt{\sum_{i=1}^{n} A_{i}^{2}} \sqrt{\sum_{i=1}^{n} B_{i}^{2}}}\end{align*}

where $\mathbf{A}$ and $\mathbf{B}$ are the vectors being compared, and $\| \cdot \|$ represents the norm operation.

Finally, the total loss function is a weighted sum of the contrastive loss and the reconstruction loss: 


(19) \begin{align*}& \mathcal{L}_{\text{total}} = \lambda_{1} \mathcal{L}_{\text{recon}} + \lambda_{2} \mathcal{L}_{\text{contr}}\end{align*}

where $\lambda _{1}$ and $\lambda _{2}$ are hyperparameters that balance the contributions of each loss term. By default, $\lambda _{1}$ and $\lambda _{2}$ are set to 0.5 and 1.0, respectively, which were determined through a grid search in the range [0.1, 1.5] with increments of 0.1, measuring performance via ARI and NMI.

### Clustering and spatial domain identification

We applied [24], a GMM-based method using the EM algorithm, to cluster latent representations and identify spatial domains. It adaptively determines the optimal cluster number for precise cellular segmentation [24]. The detailed algorithmic process is presented in Algorithm 1.

### Baseline methods

To evaluate the effectiveness of our proposed model, we compare it against multiple state-of-the-art spatial transcriptomics clustering methods, including CCST, DeepST, DiffusionST, GraphST, SEDR, SpaGCN, and STAGATE. Below is a brief introduction to each method:

- **CCST** [10]: An unsupervised graph convolutional network (GCN) clustering method that applies Deep Graph Infomax (DGI) to node embeddings.
- **DeepST** [14]: A Variational Graph Autoencoder (VGAE)-based deep learning framework that enhances the adjacency matrix by integrating morphological images, gene expression, and spatial location.
- **DiffusionST** [6]: Uses a zero-inflated negative binomial (ZINB) distribution and diffusion models to denoise and enhance spatial transcriptomics data.
- **GraphST** [9]: A self-supervised contrastive learning method that integrates GNNs with contrastive learning by minimizing embedding distances between spatially adjacent spots to obtain informative and discriminative embeddings.
- **SpaGCN** [15]: Leverages GCN-based spatial transcriptomics analysis by incorporating gene expression, spatial location, and histological images to identify spatial domains and detect spatially variable genes (SVGs) that exhibit distinct spatial expression patterns.
- **STAGATE** [7]: A graph attention autoencoder (GAT)-based framework that learns low-dimensional latent embeddings by integrating spatial information and gene expression profiles to identify spatial domains.
- **SEDR** [8]: Combines a deep autoencoder with a masked self-supervised learning strategy to construct a low-dimensional latent representation of gene expression, which is then embedded into a variational graph autoencoder (VGAE) along with spatial information for clustering.

### Evaluation metrics

#### ARI

The Adjusted Rand Index (ARI) evaluates the similarity between predicted clusters and ground truth labels, correcting for chance. It is defined as: 


(20) \begin{align*}& \text{ARI} = \frac{ \sum_{ij} \binom{n_{ij}}{2} - \frac{\sum_{i} \binom{a_{i}}{2} \sum_{j} \binom{b_{j}}{2}}{\binom{n}{2}} }{ \frac{1}{2} \left[ \sum_{i} \binom{a_{i}}{2} + \sum_{j} \binom{b_{j}}{2} \right] - \frac{\sum_{i} \binom{a_{i}}{2} \sum_{j} \binom{b_{j}}{2}}{\binom{n}{2}} }\end{align*}

Here, $n$ is the total number of samples; $n_{ij}$ is the count of samples in both predicted cluster $i$ and true cluster $j$; $a_{i}$ and $b_{j}$ are the total samples in predicted cluster $i$ and true cluster $j$, respectively.

#### NMI

Normalized Mutual Information (NMI) measures the mutual dependence between predicted and true labels: 


(21) \begin{align*}& \text{NMI} = \frac{ -2 \sum_{i=1}^{|C|} \sum_{j=1}^{K} n_{ij} \log \left( \frac{n_{ij} \cdot N}{n_{i\cdot} \cdot n_{\cdot j}} \right) }{ \sum_{i=1}^{|C|} n_{i\cdot} \log \left( \frac{n_{i\cdot}}{N} \right) + \sum_{j=1}^{K} n_{\cdot j} \log \left( \frac{n_{\cdot j}}{N} \right) }\end{align*}

$|C|$ and $K$ are the number of predicted and true clusters; $n_{ij}$ is the number of overlapping samples; $n_{i\cdot }$ and $n_{\cdot j}$ are the marginals; $N$ is the total sample count. Higher ARI and NMI values indicate better clustering alignment with ground truth.

### Implementation details

For all baseline methods, we adopted the default parameters as specified in their respective original papers. Our experiments were conducted on a single NVIDIA RTX 3060 GPU. The training process was configured for 100 epochs and a learning rate of 0.001.

## Results

### Applying SpaICL to the DLPFC dataset

**Figure 2:**
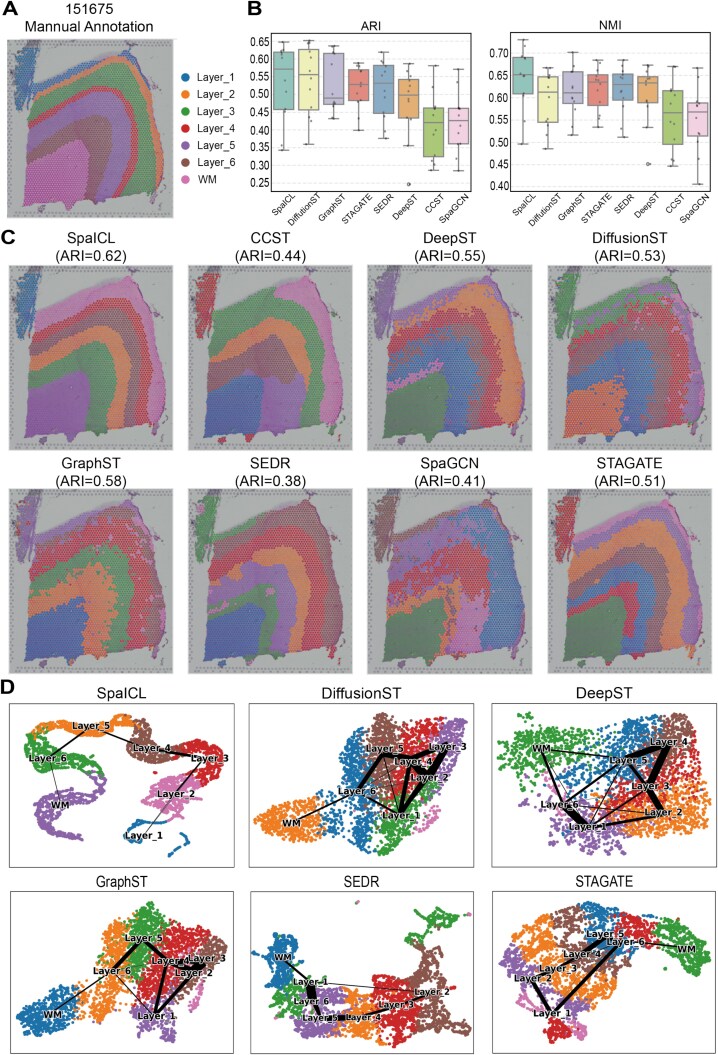
Comparison of Clustering Performance and Visualization on the DLPFC Dataset. (A) Manual annotations for slice 151675 of the DLPFC dataset. (B) Boxplots of ARI and NMI evaluation results across all 12 slices of the DLPFC dataset for various methods. (C) Example of spatial domain identification results on slice 151675 by different methods. (D) UMAP visualizations of the reduced embeddings on slice 151675 for each method.

**Performance in Clustering.** We first evaluated SpaICL on the DLPFC dataset, which includes 12 slices with manual layer annotations. SpaICL achieved the highest average ARI and NMI across all slices, along with the lowest performance variance, demonstrating strong consistency and robustness (Fig. 2B). As shown in Fig. 2C, SpaICL’s clustering results closely match the manually annotated cortical layers, particularly in boundary regions where many baseline methods produced cross-domain mixing.

To evaluate the quality of latent embeddings, we visualized the UMAP-reduced representations for slice 151675. SpaICL yielded compact, well-separated clusters that align with anatomical layers, outperforming baseline methods such as SEDR and DeepST, which showed overlapping clusters (Fig. 2D).

**Figure 3:**
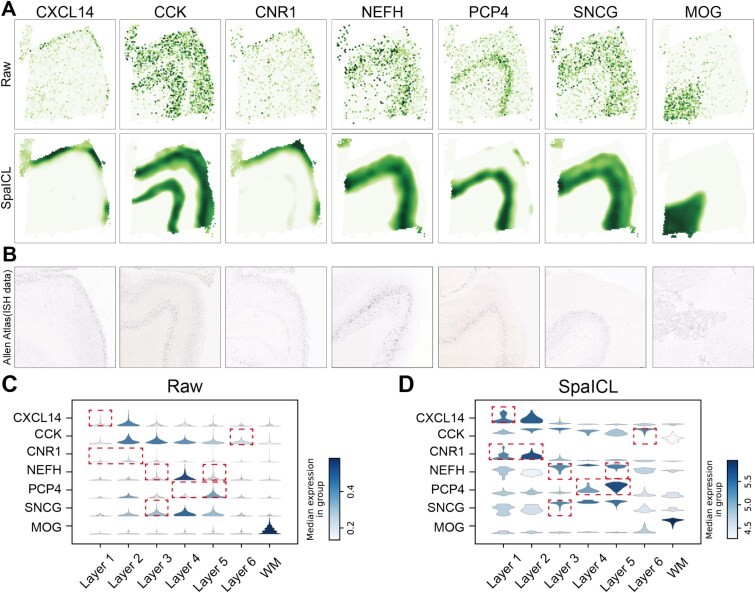
Comparison of Denoising Performance and Visualization on the DLPFC Dataset. (A) Visualizations of the raw spatial expressions and SpaICL denoised ones of six layer-marker genes in the DLPFC section 151675. (B) ISH images from cortical layer (CXCL14, CCK, CNR1, NEFH, PCP4, and SNCG) or oligodendrocyte (MOG) of the adult human brain from the Allen Human Brain Atlas. (C) Violin plots of the raw expression of layer-marker genes. (D) Violin plots of SpaICL-denoised layer-marker gene expressions, with corresponding cortical layers highlighted in red.

**Denoising Performance.** SpaICL is also capable of denoising and imputing spatial gene expression data. We applied SpaICL to denoise in the DLPFC dataset, aiming to enhance the spatial patterns of gene expression. Specifically, we focused on seven well-known layer-specific marker genes (*CXCL14*, *CCK*, *CNR1*, *NEFH*, *PCP4*, *SNCG*, *MOG*) in section 151675 and compared their original expression with the denoised results generated by SpaICL (Fig. 3A). As expected, SpaICL clearly revealed the laminar enrichment patterns of these markers. For example, *NEFH* and *SNCG* showed pronounced upregulation of gene expression in Layer 3, and *PCP4* was enriched in Layer 5, which is consistent with previously reported findings [25]. In contrast, these spatial patterns were much more diffuse and scattered in the raw data. To validate the accuracy of SpaICL’s denoising, we compared the results with in situ hybridization (ISH) data from the Allen Human Brain Atlas (Fig. 3B), which confirmed the layer-specific enrichment patterns. Furthermore, violin plots illustrate that SpaICL enhances the spatial resolution and signal strength of layer markers compared to the raw data (Fig. 3C and D), demonstrating its effectiveness in spatial denoising and enhancing the spatial patterns of layer-marker genes.

### Applying SpaICL to the BRCA dataset

**Figure 4:**
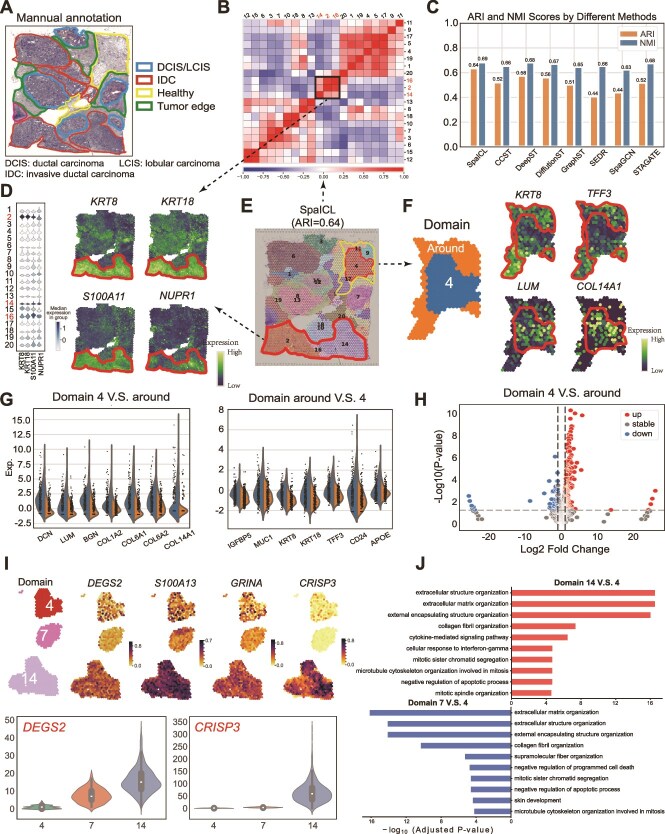
Clustering Performance and Downstream Analyses on the BRCA Dataset. (A) Manual annotations for the BRCA dataset. (B) Pearson correlation heatmap of gene expression across different spatial domains, where Domains 2, 14, and 16 exhibit significant heterogeneity compared to other domains. (C) Boxplots comparing ARI and NMI scores across different methods on the BRCA dataset. (D) Violin plots (left) and spatial visualization (right) of marker gene expression in Domains 2, 14, and 16. (E) Clustering results of the BRCA dataset using SpaICL. (F) Spatial visualization of differentially expressed genes (DEGs) between Domain 4 and its surrounding healthy tissue. (G) Violin plots of DEGs between Domain 4 and its surrounding regions. (H) Volcano plot of DEGs between Domain 4 and the surrounding healthy tissue. (I) Differential gene expression analysis (top) and violin plots (bottom) comparing Healthy (Domain 4), DCIS/LCIS (Domain 7), and IDC (Domain 14). (J) GO:BP enrichment analysis results for Domain 14 V.S. Domain 4 and Domain 7 V.S. Domain 4 comparisons.

**Performance in Clustering.** On the BRCA dataset, SpaICL achieved the highest ARI and NMI scores among all baseline methods (Fig. 4C), demonstrating robust clustering performance in heterogeneous tumor environments.

**Dissecting spatial domains at a finer level.** To assess spatial heterogeneity, we analyzed Domains 2, 14, and 16, which showed clear divergence in transcriptomic profiles from other regions (Fig. 4B). Differentially expressed genes (DEGs) such as *KRT8* and *KRT18*, associated with epithelial function [26], were highly expressed in these domains, while *NUPR1*, a prognostic marker in early-stage breast cancer [27], was upregulated (Fig. 4D). GO enrichment analysis [28] revealed enrichment in extracellular matrix organization and cytokine signaling pathways, highlighting the distinct biological roles of these regions (Fig. 4J).

Moreover, SpaICL uncovered field cancerization effects [29] by distinguishing a subregion surrounding Domain 4 (annotated as healthy). This peripheral region expressed markers such as *MUC1*, *CD24*, *TFF3*, and *IGFBP5*, which are associated with early tumorigenic changes (Fig. 4F–H) [30–33]. Meanwhile, Domain 4 expressed tumor-suppressive genes like *DCN*, *LUM*, *BGN*, and *COL1A2*, suggesting a protective microenvironment [34–37].

To explore tumor progression, we compared gene expression between healthy tissue (Domain 4), DCIS/LCIS (Domain 7), and invasive carcinoma (Domain 14). Key oncogenes such as *DEGS2*, *S100A13*, and *GRINA* showed stepwise upregulation, and *CRISP3*, linked to invasive potential [38–41], was highly expressed in Domain 14 (Fig. 4I). Pathway analysis revealed increased ECM remodeling, mitotic activity, and interferon-gamma signaling in IDC (Fig. 4J), supporting SpaICL’s capacity to delineate progressive oncogenic states.

### Applying SpaICL to the HER2+, MBA, and BCDC datasets

**Figure 5:**
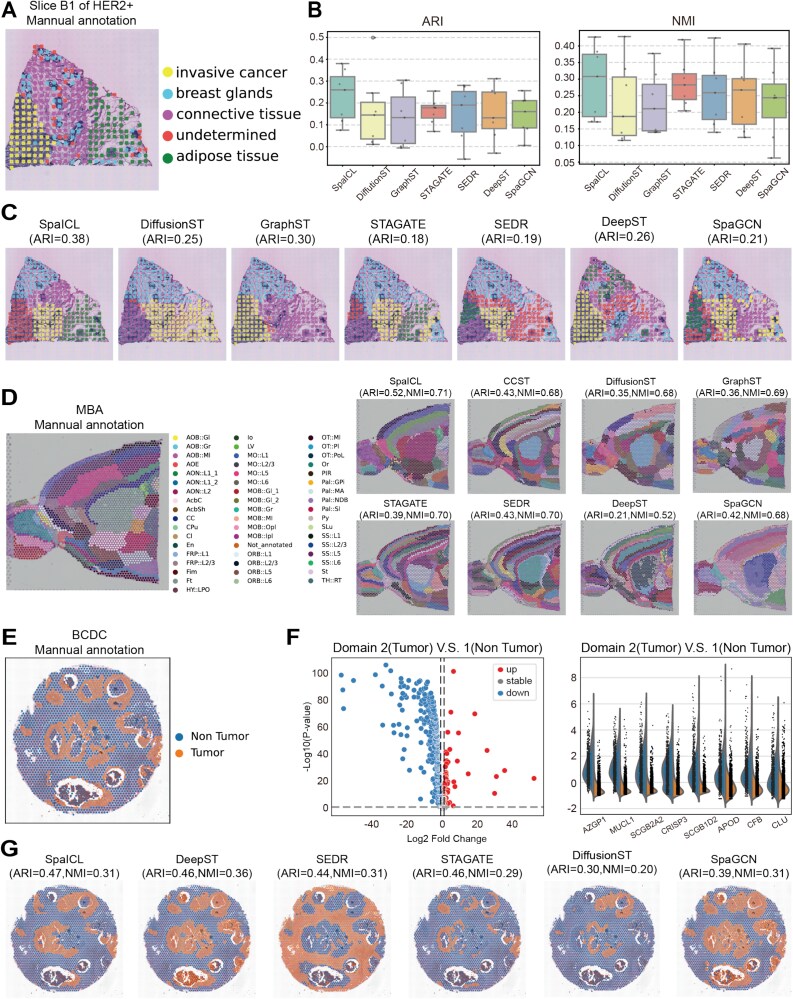
Comparison of Clustering Performance and Visualization on the HER2+, MBA, and BCDC Datasets. (A) Manual annotations for the HER2+ dataset. (B) Boxplots of ARI and NMI evaluation results across 8 slices of the HER2+ dataset for various methods. (C) Example of spatial domain identification results on slice B1 of the HER2+ dataset by different methods. (D) Manual annotations for the MBA dataset (left), along with a comparison of spatial clustering results, ARI, and NMI (right). (E) Manual annotations for the BCDC dataset. (F) Volcano graph of DEGs between Domains 2(tumor) and 1(non tumor)(left), along with the analysis of most differentially expressed genes (right). (G) Spatial clustering results from the top 6 performing methods, along with a comparison of ARI and NMI.

To further validate the robustness and generalizability of our method, we applied SpaICL to three additional datasets: HER2+ (multi-slice dataset), MBA (single-slice dataset), and BCDC (single-slice dataset) (Fig. 5).

**HER2+ Dataset.** On the HER2+ dataset, which consists of eight slices, SpaICL was the only method capable of clearly distinguishing the invasive cancer, connective tissue, and adipose tissue regions in the B1 slice (Fig. 5A, C). These regions were well-separated and hierarchically organized, demonstrating SpaICL’s superior ability to capture fine-grained spatial structures. In comparison, other baseline methods failed to achieve such precise delineation. Quantitatively, SpaICL also outperformed other methods in terms of ARI and NMI scores across the dataset (Fig. 5B).

**MBA Dataset.** On the MBA dataset (Fig. 5D), SpaICL significantly outperformed other methods in ARI and NMI. Additionally, SpaICL successfully identified and distinctly separated the granular cell layer (Gr layer) of the main olfactory bulb (highlighted by the red circle in Fig. 5D) from surrounding tissues. This region exhibits clear morphological differences from adjacent brain areas in histological images, demonstrating SpaICL’s superior spatial resolution and clustering precision.

**BCDC Dataset.** The BCDC dataset is a single-section dataset that provides only a binary annotation of “Tumor vs. Non-Tumor”. To further explore the biological relevance of our clustering results, we conducted a DEGs analysis between the identified tumor and non-tumor regions. In this study, we highlight the most significant differentially expressed genes (Fig. 5E and F). Among them, *AZGP1* and *CRISP3* are associated with breast cancer invasiveness [41], while *SCGB2A2* and *SCGB1D2* serve as common markers for disseminated tumor cells (DTCs) [42]. Additionally, *MUCL1* is recognized as an attractive tumor-associated antigen and a potential therapeutic target [43]. We further visualized the clustering results of the top six performing methods based on ARI and NMI (Fig. 5G). Notably, SpaICL consistently achieved the best performance, demonstrating its superior ability to delineate tumor heterogeneity in spatial transcriptomics data.

**Table 2:** Ablation study on the impact of different modules of SpaICL. We used the median value of ARI and NMI as the value for multi-slice datasets, DLPFC and HER2+

**Ablation Type**	**DLPFC**		**BRCA**		**MBA**		**HER2+**		**BCDC**	
	**ARI**	**NMI**	**ARI**	**NMI**	**ARI**	**NMI**	**ARI**	**NMI**	**ARI**	**NMI**
w/o CLM	0.527$\pm $0.012	0.642$\pm $0.009	0.563$\pm $0.005	0.652$\pm $0.008	0.472$\pm $0.013	0.676$\pm $0.007	0.200$\pm $0.017	0.250$\pm $0.020	0.362$\pm $0.011	0.243$\pm $0.026
w/o Image	0.531$\pm $0.006	0.634$\pm $0.011	0.563$\pm $0.005	0.652$\pm $0.007	0.494$\pm $0.018	0.660$\pm $0.008	0.196$\pm $0.009	0.249$\pm $0.025	0.378$\pm $0.020	0.248$\pm $0.014
w/o $\mathcal{L}_{\text{contr}}$	0.505$\pm $0.005	0.633$\pm $0.005	0.570$\pm $0.012	0.656$\pm $0.009	0.474$\pm $0.015	0.671$\pm $0.009	0.213$\pm $0.022	0.258$\pm $0.020	0.356$\pm $0.006	0.266$\pm $0.017
**SpaICL**	**0.566$\pm $0.010**	**0.660$\pm $0.011**	**0.613$\pm $0.023**	**0.674$\pm $0.008**	**0.513$\pm $0.011**	**0.704$\pm $0.006**	**0.234$\pm $0.026**	**0.266$\pm $0.030**	**0.434$\pm $0.044**	**0.285$\pm $0.039**

### Ablation study

To assess the contribution of key components in SpaICL, we conducted an ablation study by removing specific modules and measuring performance across multiple datasets (Table 2). The tested variants were: (1) w/o CLM—removing the Curriculum Learning Module and using direct global readout; (2) w/o Image—excluding the image modality and using only gene expression; (3) w/o $\mathcal{L}_{\text{contr}}$—discarding the contrastive loss and training with reconstruction loss only; (4) w/o UNI—replacing our image feature encoder UNI with standard ImageNet-pretrained models (ResNet/VGG/DenseNet/Inception), as detailed in (Supplementary Table S2). Results show consistent performance drops in all cases, confirming that each module contributes significantly to SpaICL’s effectiveness, with the full model achieving the best ARI and NMI scores.

### Time cost

We also evaluated the runtime efficiency of SpaICL compared to baseline methods. As shown in Table 3, our proposed SpaICL achieves the lowest time cost (12.68 seconds) on the BRCA dataset. It outperforms SEDR (14.25 s) and SpaGCN (28.07 s), and is significantly faster than DeepST and DiffusionST, both of which require over 10 minutes. These results demonstrate the superior computational efficiency of SpaICL.

**Table 3:** Time cost of different methods on the BRCA dataset

**Method**	**Time cost**
SEDR [8]	14.25 s
CCST [10]	46.87 s
SpaGCN [15]	28.07 s
STAGATE [7]	32.87 s
GraphST [9]	27.97 s
DeepST [14]	>10 min
DiffusionST [6]	>10 min
SpaICL (Ours)	**12.68 s**

## Conclusion and discussion

In this work, we proposed SpaICL, an image-guided curriculum strategy-based graph contrastive learning framework for clustering spatial transcriptomics data. By integrating gene expression profiles, spatial coordinates, and histological images, SpaICL constructs a unified low-dimensional representation that enables accurate and biologically meaningful spatial domain identification across diverse tissue types.

SpaICL introduces three key innovations: (1) a complementary masking strategy for generating diverse gene expression views to enhance contrastive learning; (2) a dual cross-attention mechanism for deep alignment and fusion of morphological and transcriptomic features; and (3) a curriculum learning paradigm that gradually incorporates neighborhood information to improve training stability and multiscale sensitivity. Together, these components address core challenges in ST analysis, including over-smoothing, modality misalignment, and feature redundancy.

SpaICL achieves consistently superior clustering accuracy (ARI and NMI) compared to state-of-the-art approaches across five benchmark spatial transcriptomics datasets, highlighting its strong robustness and generalization capability in capturing biologically meaningful spatial patterns.

While SpaICL demonstrates robust performance across diverse datasets, two limitations warrant consideration: (1) The framework’s effectiveness depends on high-quality histology images, as performance may degrade with low-resolution or suboptimally stained tissue sections. (2) The computational cost of the Unified Neighborhood Integration (UNI) module scales with dataset size, though this is partially mitigated through GPU acceleration strategies. These constraints highlight practical considerations for applications involving large-scale or image-challenged datasets.

To broaden the impact of SpaICL, we envision three key extensions: (1) Adaptation to single-cell resolution spatial technologies (e.g. MERFISH or seqFISH+) to resolve finer-grained biological structures. (2) Integration of temporal dynamics for developmental and disease progression studies, enabling 4D spatial-transcriptomic analysis. (3) Development of lightweight UNI alternatives (such as distilled architectures or attention sparsification) to enhance accessibility for resource-constrained research settings. These advancements would further establish SpaICL as a versatile framework for next-generation spatial omics research.

**Key Points**

- SpaICL combines multi-modal information including histology image, spot-based spatial transcriptomics data and spatial information to accurately identify spatial domains in spatial transcriptomics data.
- SpaICL employs a curriculum learning strategy, progressively aligning local before global information, which stabilizes multi-modal training and improves convergence.
- A dual cross-attention fusion module is designed to integrate histological characteristics and gene embeddings, enabling fine-grained spatial and morphological alignment.
- Extensive experiments on five spatial transcriptomics datasets demonstrate that SpaICL consistently outperforms existing methods in both clustering performance and biological interpretability.

## Supplementary Material

SpaICL_Supplementary_Materials_bbaf433

## Data Availability

The five benchmark ST datasets used in this study are publicly available as follows: DLPFC (human dorsolateral prefrontal cortex) dataset: (http://spatial.libd.org/spatialLIBD); MBA (anterior mouse brain tissue), BCDC (human breast cancer: in situ ductal carcinoma and invasive carcinoma) and BRCA (human breast cancer) datasets: (https://support.10xgenomics.com/spatial-gene-expression/datasets); HER2+ dataset (HER2-positive breast tumors): (https://github.com/almaan/her2st?tab=readme-ov-file).
