# Supplementary material for: SpaICL: image-guided curriculum strategy-based graph contrastive learning for spatial transcriptomics clustering
Source: Brief Bioinform. 2025 Aug 21;26(4):bbaf433. doi: 10.1093/bib/bbaf433 (PMC12368861; doi:10.1093/bib/bbaf433)
Supplement: SpaICL_Supplementary_Materials_bbaf433 [file spaicl_supplementary_materials_bbaf433.pdf]

Supplementary Materials for

SpaICL: Image-Guided Curriculum Strategy-Based Graph Contrastive Learning for Spatial Transcriptomics Clustering

Supplementary Table

Table S1. Ablation test on the BRCA dataset using different clustering methods.

Method\Seed	568		708		1262		470		463		Average	
	ARI	NMI	ARI	NMI	ARI	NMI	ARI	NMI	ARI	NMI	ARI	NMI
K-means	0.4542	0.5686	0.4818	0.5775	0.4831	0.5817	0.487	0.5801	0.4257	0.5623	0.4664±0.0234	0.5740±0.0074
Spectral	0.0109	0.1278	0.0114	0.1138	0.0113	0.1144	0.0109	0.1278	0.0109	0.1278	0.0111±0.0002	0.1223±0.0067
**Mclust**	**0.6072**	**0.6724**	**0.6383**	**0.6864**	**0.5967**	**0.6813**	**0.579**	**0.6697**	**0.6395**	**0.6851**	**0.6121±0.0236**	**0.6790±0.0067**

Table S2. Ablation study on image embeddings produced by different platforms. We used the median value of ARI and NMI as the value for multi-slice datasets, DLPFC and HER2+.

Method\Dataset	DLPFC		BRCA		MBA		HER2+		BCDC	
	ARI	NMI	ARI	NMI	ARI	NMI	ARI	NMI	ARI	NMI
ResNet50	0.533±0.013	0.633±0.015	0.562±0.012	0.659±0.010	0.451±0.012	0.686±0.005	0.201±0.017	0.249±0.018	0.332±0.026	0.199±0.025
Resnet152	0.522±0.013	0.629±0.017	0.573±0.014	0.663±0.005	0.463±0.015	0.686±0.009	0.210±0.006	0.249±0.021	0.368±0.069	0.250±0.054
Vgg19	0.529±0.013	0.639±0.009	0.571±0.025	0.664±0.011	0.468±0.015	0.689±0.007	0.206±0.014	0.249±0.019	0.350±0.027	0.250±0.029
Vgg16	0.523±0.024	0.640±0.009	0.568±0.013	0.669±0.004	0.461±0.004	0.683±0.004	0.225±0.015	0.255±0.032	0.343±0.035	0.232±0.051
DenseNet121	0.527±0.011	0.633±0.008	0.571±0.022	0.667±0.010	0.476±0.017	0.690±0.008	0.208±0.011	0.251±0.040	0.361±0.064	0.240±0.051
Inception_v3	0.518±0.017	0.635±0.011	0.597±0.018	0.674±0.010	0.462±0.016	0.683±0.006	0.174±0.013	0.243±0.045	0.361±0.064	0.240±0.051
**UNI**	**0.566±0.010**	**0.660±0.011**	**0.613±0.023**	**0.674±0.008**	**0.513±0.011**	**0.704±0.006**	**0.234±0.026**	**0.266±0.030**	**0.434±0.044**	**0.285±0.039**

Supplementary Figures

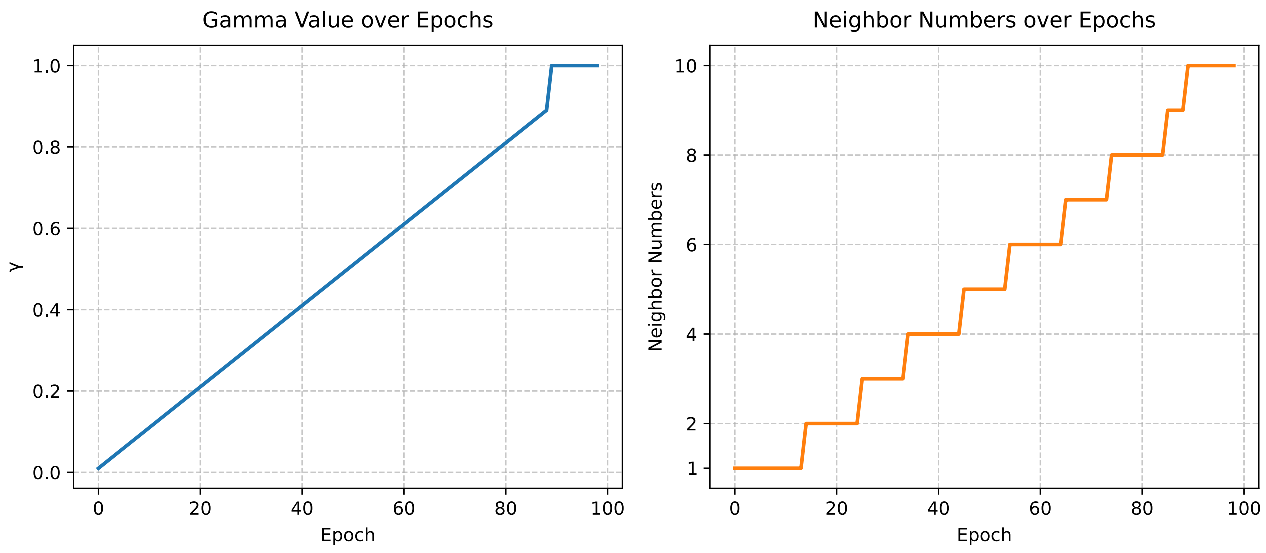

Figure S1. Evolution of Gamma and Neighbor Numbers over 100 Training Epochs (with $Number_{neighbor}$=10). Evolution of the $\gamma(t)$ value across t(training epochs) (left); Derived neighbor counts computed as $max(1, \gamma \times Number_{neighbor})$, showing how the interaction radius adapts during learning (right).

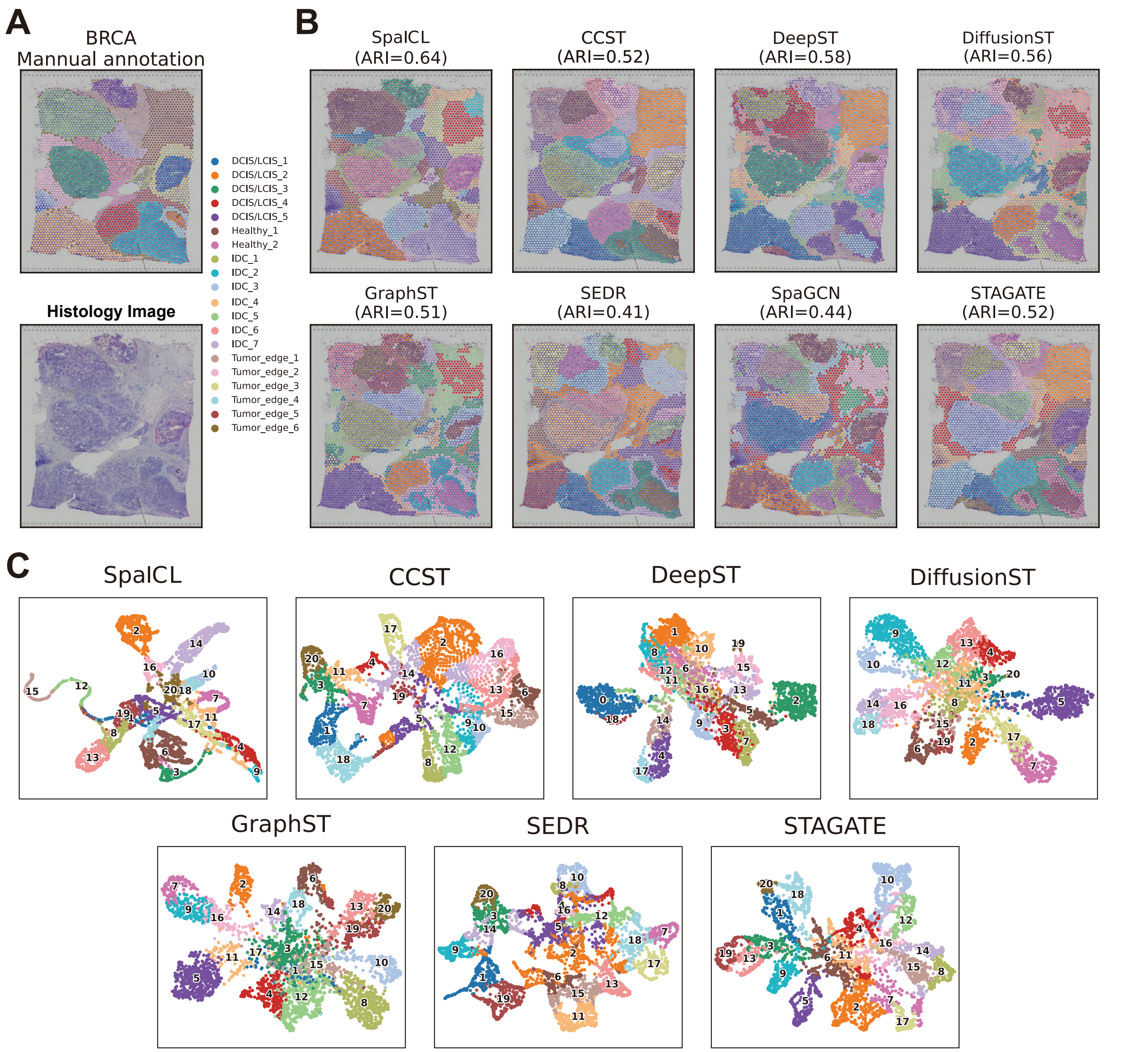

Figure S2. SpaICL identifies spatial domains on the BRCA (human breast cancer) dataset. (**A**) Manual annotation layer structure and the histology image for BRCA dataset. (**B**) Spatial domains are detected by SpaICL, CCST, DeepST, DiffusionST, GraphST, SEDR, SpaGCN, and STAGATE. (**C**) The UMAP visualization of the experimental results.

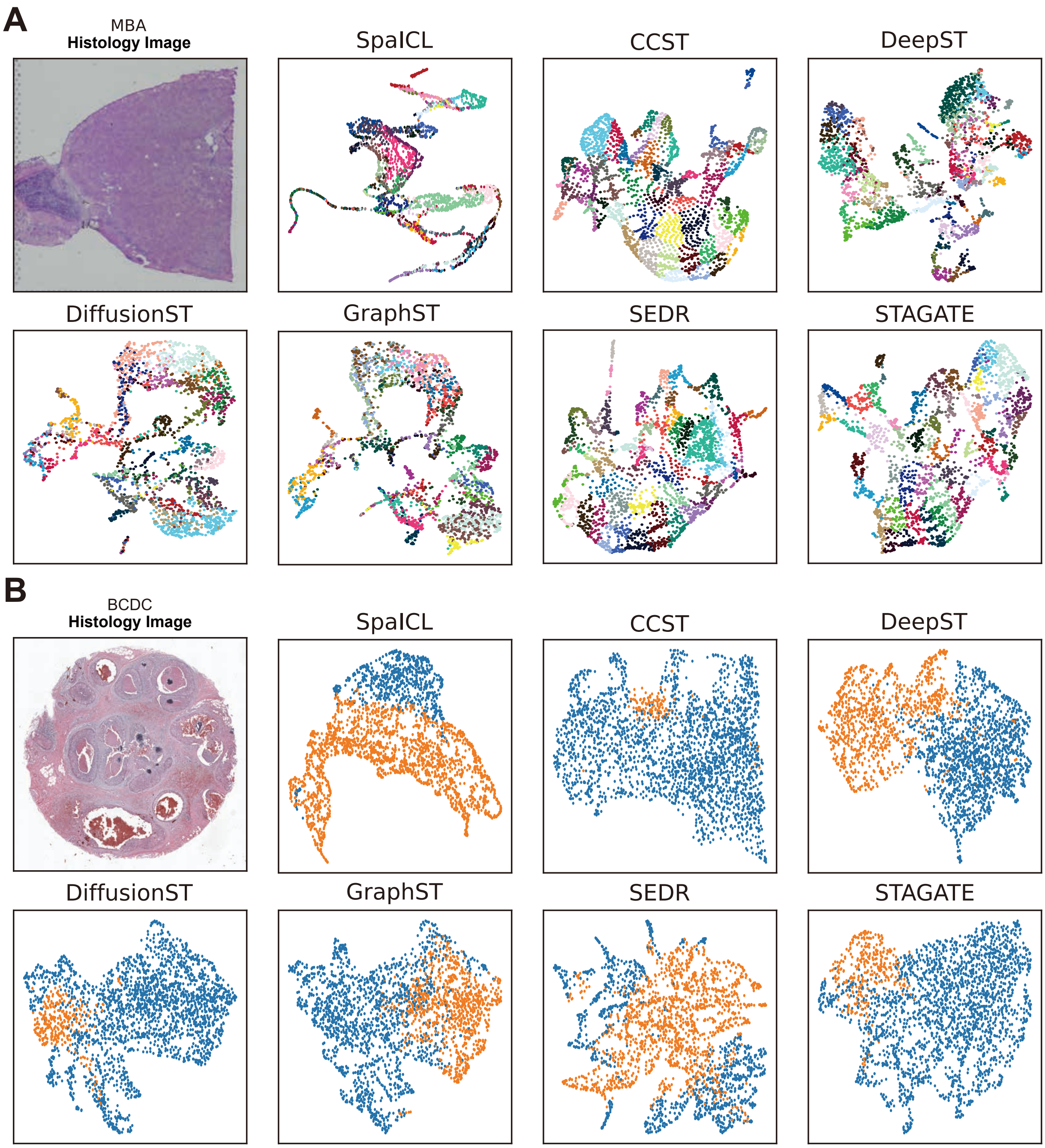

Figure S3. Spatial domains are detected by SpaICL, CCST, DeepST, DiffusionST, GraphST, SEDR, and STAGATE on the MBA (anterior mouse brain tissue) dataset and BCDC (human breast cancer: in situ ductal carcinoma and invasive carcinoma) dataset. (**A**) Histology image and UMAP visualization of the experimental results for BRCA dataset. (**B**) Histology image and UMAP visualization of the experimental results for BCDC dataset.
